# Comprehensive peace agreement implementation and reduction in neonatal, infant and under-5 mortality rates in post-armed conflict states, 1989–2012

**DOI:** 10.1186/s12914-015-0066-7

**Published:** 2015-10-08

**Authors:** Madhav Joshi

**Affiliations:** Kroc Institute for International Peace Studies, University of Notre Dame, 331 Hesburgh Center, Notre Dame, IN 46556-5677 USA

**Keywords:** Comprehensive peace agreement, Child mortality, Conflict termination, Post-armed conflict, Negotiation, Equity, MDG 4

## Abstract

**Background:**

Previous studies suggest that countries with a higher child mortality rate are more susceptible to armed conflict onset as well as recurrence. Studies do not explain conditions under which child mortality declines among post-armed conflict states. This article argues that where armed conflict is terminated through negotiation and implementation of comprehensive peace agreements (CPAs), the child mortality rate declines. This is due to the implementation of provisions in CPAs, which addresses underlying grievances of the poor, underserved and marginalized segments of the population, as well as the successful reconstruction of the health sector. CPA implementation resolves hostilities between armed rivals and facilitates the return process for internally displaced persons and refugees. The CPA implementation may also encourage the return of aid workers and health professionals to conflict-affected zones.

**Method:**

This study utilizes new data on CPA implementation and examines neonatal, infant and under-5 mortality rates among 73 post-armed conflict countries between 1989 and 2012. Multivariate cross-sectional time series correlation (fixed effect) methods are used to analyze the data.

**Results:**

Within post-armed conflict states, a decline in neonatal, infant and under-5 mortality rates is associated with higher CPA implementation rates. Additionally, this study shows that higher GDP per capita, higher levels of democracy, and more primary school enrollment are also associated with lower child mortality rates. On the other hand, child mortality rates increase following a rebel victory in armed conflict.

**Conclusion:**

Ongoing armed conflicts are responsible for massive displacements and the destruction of economic, healthcare and human infrastructure, thus hindering improvements in child survival. For better health outcomes in post-armed conflict countries, ongoing armed conflict must cease through the signing and implementation of a CPA. Short-term and long-term public health issues are discussed in concluding comments.

**Electronic supplementary material:**

The online version of this article (doi:10.1186/s12914-015-0066-7) contains supplementary material, which is available to authorized users.

## Background

Reducing child mortality rates by two-thirds from 1990 to 2015 is one of the main objectives of the Millennium Development Goals (MDG 4).^1^ Progress has fallen short, however, despite the substantial support from various international development forums, donor agencies, and political leaders influential in both international and domestic politics [1–6]. According to the 2014 MDG progress report (p. 25), the goal of reducing child mortality by two-thirds from 1990 levels will take an additional 15 years, that is, until 2028 [2]. The report suggests that Sub-Saharan African countries in particular, many of which have gone through cycles of armed conflict, are the laggards (p. 26). In fact, the United Nations International Children’s Fund (UNICEF) suggests that eight out of the ten countries with the highest child mortality rates are either in armed conflict or considered to be in politically unstable situations [3]. These recent UNICEF and MDG reports highlight the importance of political stability, noting that reducing child mortality depends on states’ abilities to end the cycle of armed conflict and transition out of political fragility.

A reduction in infant mortality is one of the indicators of the overall performance of the health sector, a sector highly influenced by the policy choices the political regime makes. Studies show that democracies consistently have higher levels of public service provisions (i.e. health education) than autocracies [7]. One of the reasons could be that the ruling party’s chance to win elections in democracies depends on the successful provision of public goods and services [8–10]. Regardless of the level of per capita gross domestic product (GDP), the child mortality rate is significantly lower in democracies than in autocracies for the reasons mentioned above [8, 11, 12].

In the past decade, studies have established that armed conflicts cause breakdowns in countries’ healthcare systems [13–17]. Such breakdowns lead to limited or no access to healthcare facilities. In many instances, postnatal care is nonexistent because trained healthcare professionals are forced to leave the communities they serve. While overall child mortality rates have declined in countries undergoing armed conflict due to shrinking costs of war, low cost health interventions during peacetime, humanitarian interventions, immunization and localized nature of armed conflict [18], child mortality is consistently shown to be significantly higher among countries undergoing armed conflict [19]. Nicaragua and Eastern Myanmar demonstrate two realities of child mortality and armed conflict. The outbreak of armed conflict in Nicaragua stalled a trend of declining infant mortality rates [13], and Eastern Myanmar, an area affected by ongoing ethnic conflicts, has a high level of infant mortality [16, 20]. Such negative effects on the public health sector do not disappear immediately after conflict termination. Public health and social science studies have reported that conflict-related deaths and injuries are likely to be observed even after the conflicts are terminated for reasons related to a reduced economic capacity to support public health sector in a post-war context [15, 21–23]. Other reasons for a higher conflict related deaths and injuries in a post-war period are related to interruptions in access to basic services, availability of healthcare professionals, and conflict caused environmental damages that could give rise to disease and ill health. While studies suggest the significant impact of deaths and disabilities related to armed conflict [24], most of these studies do not address the conditions under which societies emerging from the cessation of conflict are performing better in reducing child mortality rates. This study addresses this gap in scholarship by examining the rates of neonatal, infant, and under-5 mortality in relation to the negotiation and implementation of comprehensive peace agreements in 73 post-armed conflict countries between 1989 and 2012. In this study, a post-armed conflict country is a country where at least one conflict or one conflict dyad is coded “terminated” in the Uppsala Conflict Data Program (UCDP) conflict termination data [25].^2^

### Pattern in neonatal, infant, and under-5 mortality rates

The 2014 MDG annual progress report and other earlier studies suggest a consistent pattern of decline in the infant mortality rate [3, 5, 26]. According to the World Bank data [27], over the last two decades (between 1989 and 2012), global neonatal deaths declined by 11.7 %, infant mortality by 26.4 %, and under-5 mortality by 39.7 %. Post-armed conflict countries follow the global pattern despite having relatively high mortality rates, but show a slower decline in the neonatal and infant mortality rates. For post-armed conflict countries in the same time period, the neonatal death rate declined by 9.47 %, infant mortality by 24.93 %, and under-5 mortality by 42.17 %. However, two things remain unaddressed. First, post-armed conflict countries are consistently behind the global average in terms of child mortality rates. Second, significant variation exists among post-armed conflict countries, with some countries retaining high rates of child mortality and others seeing a decline in child mortality following conflict.

One reason for this variation is seen in the differences of conflict termination – whether the conflict ends through rebel victory, government victory, or negotiation and implementation of comprehensive peace agreements (CPAs). Between 1989 and 2012, for example, neonatal deaths declined by 12.19 % among post-armed conflict countries that negotiated and implemented CPAs, 2.7 % among countries with rebel victories, and 0.09 % in countries with government victories. The infant mortality rate also showed significant variation. Infant mortality declined by 40.64 % in countries with CPAs, 14.63 % in countries with a rebel victory, and increased by 1.46 % in countries with a government victory. The pattern of under-5 mortality rate follows, with a decline of 70.01 % for countries with CPAs, 16.82 % for the countries with rebel victories, and an increase of 4.47 % for countries with a government victory. As these rates suggest, post-armed conflict states that negotiated and implemented comprehensive peace agreements consistently performed better in reducing child mortality rates than those that did not.

This study explains child mortality decline among post-armed conflict countries by examining the negotiation and implementation of CPAs. The Peace Accords Matrix (PAM) project defines a CPA as the final agreement negotiated to end intrastate armed conflict that involves (a) major conflict actors in the negotiation process, and (b) substantive issues included in the negotiation [28, 29]. Using this definition, the project identifies 34 CPAs negotiated in 31 countries between 1989 and 2012. The project identifies 51 different provisions in a CPA, categorized under six different clusters. Most of the provisions are designed to provide more access for excluded groups (rebel groups and their constituencies) to state power and resources. This access is increased by implementing reforms in government institutions and the security sector, guaranteeing rights to minorities, women and children, and ensuring the socio-economic development of impoverished regions (focusing on infrastructure building and improving the delivery of public services).

Implementation of reforms outlined in CPAs can directly impact child mortality rates. Child mortality rates are disproportionately high in armed conflict countries because of the destruction of healthcare infrastructure (including hospitals and healthcare facilities), security threats to health professionals, and the displacement of populations and health service providers. In conflict zones, health delivery is often politicized and difficult to provide effectively due to the fear of reprisals by rebels or state forces. After the CPA is signed, however, armed hostility may cease, thus facilitating the return of internally displaced persons (IDPs) and refugees. Implementing security sector-related reforms in CPAs might mitigate future violence and increase the chance of a lasting ceasefire [29]. As a result of an improved security environment, health professionals and aid workers may also be encouraged to return.

By implementing CPA provisions, post-armed conflict countries can also improve institutional capacities which may increase an excluded group’s access to state power and resources. Implementing socio-economic provisions in CPAs may help to rebuild damaged or destroyed infrastructure, and improve service delivery in marginalized or excluded areas. Marginalized populations tend to support rebel uprisings, so addressing their grievances is critical to avoid future conflict. In Mozambique, for example, the government prioritized the rehabilitation of the health network and return of health services to previously closed and underserved areas under the control of the rebel group, RENAMO (Resistência Nacional Moçambicana) [30].

All governments respond to the needs of the poor and marginalized segments of the population to varying degrees. However, post-armed conflict governments face a particular challenge. These governments have to make sure that the underlying grievances of the marginalized segments of the population are addressed so that there is no incentive for them to join rebel movements or return to violence. The government has to demonstrate its commitment to a sustainable peace, and the best way to demonstrate its commitment to peace is through implementing provisions negotiated in the CPA. Therefore, countries that negotiate and implement CPAs at a higher rate will be more likely to improve health infrastructure, decrease security threats for health workers, increase opportunities for the return of IDPs and refugees, and increase institutional capacities. All of these factors lead to a declining child mortality rate. In countries where parties did not sign CPAs, inequality persists and grievances of those without access to power or resources mostly remain unaddressed. A decisive government or rebel victory may still lead to a decline in child mortality rates if the post-armed conflict government successfully eliminates the threat of violence, prioritizes the rebuilding of hospitals and health posts shattered during the conflict, and incentivizes healthcare professionals to return to work. These one-sided victories (government or rebel), however, may lead the party in power to focus on a security buildup at the expense of delivering services, as seen in post-2009 Sri Lanka. The victorious side (either government or rebel) may also support exclusionary policies as a way to safeguard their constituencies’ interests.

## Method

### Post-armed conflict cases

Empirical analysis includes 73 post-armed conflict countries with 173 conflict terminations between 1989 and 2012.^3^ An additional file shows list of post-armed conflict countries [see Additional file 1]. This sample includes 31 countries that negotiated and implemented CPAs. Post-armed conflict countries are identified by using the armed conflict data from the Uppsala Conflict Data Program [31].

The dependent variable is the natural log transformation of annual neonatal, infant, and under-5 mortality rates using data from the World Bank’s World Development Indicators [27]. Distributions in the mortality rates data are more likely to be skewed and therefore the natural log transformation is used.

Multivariate cross-sectional time series correlation (fixed effect) methods are used to analyze the data. The main explanatory variable is the comprehensive peace agreement (CPA) implementation rate, which provides insights on the fulfillment of peace agreement commitments such as ceasing hostilities, institutional and security sector reforms, guarantees of various rights, and socio-economic development measures [29]. In the data, the level of implementation is coded by using the ordinal measure: “no implementation” (coded “0”), “minimum” (coded “1”), “intermediate” (coded “2”), or “full” (coded “3”), for each provision for the first ten years of implementation.^4^ These ordinal implementation scores for the negotiated provisions are used to generate a CPA implementation rate variable. The implementation rate is derived from dividing the sum of the actual implementation value for all provisions by the highest possible score for implementing all provisions in the CPA, and multiplying the results by 100. For example, Liberia’s 2003 Accra Peace Agreement has 27 different provisions. If every provision in the accord was fully implemented in 2003, the highest possible sum of the implementation value would be 81 or (27*3). The sum of the actual implementation value is 34, which therefore yields a rate of 41.98 ((34/81)*100). For all of the CPA cases that marked the tenth year of implementation in 2002, the implementation rate at year 10 was carried over until 2012. For countries that did not sign a CPA, this variable is coded “0.”

Given the collaborative nature of conflict resolved through peace agreements, one may assume health needs are prioritized as a way to address grievances surrounding inequalities. A decisive victory may also lead to positive health benefits, because while the group in power may prioritize other issues, like general economic development and welfare policies, these policies may also lead to lower infant mortality rates. Therefore, different types of conflict termination are controlled in the analysis [25]. Using conflict termination data, four binary variables are generated in the analysis: terminated in a peace agreement, terminated in a government victory, terminated in a rebel victory, and low activity. “Low activity” serves as the reference category variable.^5^ Similarly, no matter the types of conflict termination, healthcare system recovery is likely to take significant time. Improvement in the healthcare system may be more noticeable a few years after termination since improvement will require initiation and implementation of related reforms in the post-conflict years. To capture this time lag, the analysis controls for the first 5 years of peace. This variable captures time for things to settle out once the conflict terminates. This variable is coded as a binary variable.

Since previous studies suggest a negative effect of ongoing armed conflict on overall future health performance [19, 21–24], the analysis controls for ongoing conflict with a binary variable and data from the Uppsala Conflict Data Program [32]. The same data source is used to control for the duration of the conflict. The severity of the conflict can have lingering effects on public health [21, 23] and therefore, the cumulative deaths variable (derived from the lowest estimate of battle related deaths from the UCDP data) is used in the analysis as a control for conflict severity and its effects on the population [32, 33]. Physical healthcare infrastructure can also be the target of a military campaign in territorial conflicts, if healthcare facilities are used as sanctuaries or if service deliveries are controlled by the rebel groups. As a result, child mortality patterns might be different in territorial conflicts compared to other types of conflict. Therefore, the analysis includes a control for territorial conflict with a binary variable [31].

Prior studies have suggested that a democratic regime has better redistributive policies than a non-democratic regime, with variation depending on income levels [7–11]. Analysis performed in this study controls for democracy by using the executive constraints (XCONST) variable in Polity 2 data [34]. Secondly, a higher national income is significantly related to a lower infant mortality rate [35]. Therefore, GDP per capita (at a constant 2005 value) is controlled in the analysis by using the World Bank data [27]. An increase in educational attainment is also associated with a decline in child mortality [21, 23, 36, 37]. The World Bank data [27] is therefore used to control for overall primary school enrollment. Government expenditure on healthcare is also controlled for with data from the World Bank [27]. All of these variables are lagged by a year. Descriptive statistics for variables used in analysis are presented in Table 1.

**Table 1:** Descriptive statistics

Variable	Obs.	Mean	Std. Dev.	Min.	Max.
Neonatal mortality rate (log)	1351	3.063	0.732	0.742	4.072
Infant mortality rate (log)	1359	3.685	0.866	1.194	5.109
Under-5 mortality rate (log)	1359	3.980	0.979	1.435	5.745
CPA implementation rate_t-1_	1359	21.707	33.921	0.000	95.830
Rebel victory_t-1_	1287	0.082	0.275	0.000	1.000
Government victory_t-1_	1287	0.148	0.356	0.000	1.000
Peace accord_t-1_	1287	0.430	0.495	0.000	1.000
Ongoing conflict_t-1_	1359	0.363	0.481	0.000	1.000
Territorial conflict	1360	0.324	0.468	0.000	1.000
GDP per capita ×1000 (2005)_t-1_	1283	2.998	5.754	0.500	40.452
Executive Constraint_t-1_	1359	0.503	0.500	0.000	1.000
Primary enrollment (100,000)_t-1_	1088	61.961	175.924	0.306	1400.000
First 5 years of peace	1360	0.477	0.500	0.000	1.000
War duration in months	1353	61.232	135.247	0.033	1568.121
Cumulative deaths in conflict × 1000	1354	15.590	35.605	0.020	235.666
Health Exp.(% Govt. Spending)t_t-1_	1102	9.838	4.719	0.000	42.378

## Results and discussion

Table 2 presents results from three different model specifications: neonatal mortality, infant mortality, and under-5 mortality. In the first models for each group, the CPA implementation rate is used with three different armed conflict-related variables: war duration in days, cumulative deaths in conflict, and whether the conflict was territorial. Model 2 excludes war duration in days and cumulative deaths in conflict, but includes additional armed conflict-related variables (e.g. government victory, rebel victory) and controls for executive constraints, GDP per capita, and primary school enrollment. Model 3 is the full model that includes all armed conflict-related variables introduced in Models 1 and 2 along with government expenditure on the health sector and the first 5 years of peace variables.

**Table 2:** Child mortality in post-conflict countries

	Neonatal			Infant			Under-5		
	(1)	(2)	(3)	(1)	(2)	(3)	(1)	(2)	(3)
CPA implementation rate_t-1_	−0.003^***^	−0.001^***^	−0.001^*^	−0.004^***^	−0.002^***^	−0.001^**^	−0.005^***^	−0.002^***^	−0.002^**^
	(0.000)	(0.000)	(0.000)	(0.000)	(0.000)	(0.000)	(0.000)	(0.000)	(0.001)
War duration in months	0.000		0.000	0.000		0.000	0.000		−0.000
	(0.000)		(0.000)	(0.000)		(0.000)	(0.000)		(0.000)
Cumulative deaths in conflict × 1000	−0.017^***^		−0.010^***^	−0.021^***^		−0.011^***^	−0.023^***^		−0.012^***^
	(0.002)		(0.002)	(0.002)		(0.002)	(0.002)		(0.003)
Territorial Conflict	0.031	−0.041	−0.051	0.047	−0.034	−0.065	0.071^*^	−0.017	−0.051
	(0.024)	(0.026)	(0.033)	(0.030)	(0.032)	(0.042)	(0.033)	(0.036)	(0.047)
Rebel victory_t-1_		0.197^***^	0.195^***^		0.308^***^	0.306^***^		0.358^***^	0.357^***^
		(0.047)	(0.053)		(0.058)	(0.066)		(0.065)	(0.074)
Government victory_t-1_		−0.031	−0.036		−0.031	−0.041		−0.031	−0.044
		(0.030)	(0.028)		(0.037)	(0.035)		(0.041)	(0.040)
Peace accord_t-1_		0.033	0.007		0.052	0.016		0.044	−0.005
		(0.022)	(0.023)		(0.028)	(0.029)		(0.031)	(0.032)
Ongoing conflict_t-1_		0.091^***^	0.053^***^		0.117^***^	0.069^***^		0.134^***^	0.081^***^
		(0.015)	(0.016)		(0.019)	(0.020)		(0.021)	(0.022)
GDP per capita in 1000 (2005)_t-1_		−0.079^***^	−0.091^***^		−0.091^***^	−0.104^***^		−0.093^***^	−0.106^***^
		(0.005)	(0.006)		(0.006)	(0.007)		(0.007)	(0.008)
Executive Constraint_t-1_		−0.127^***^	−0.091^***^		−0.151^***^	−0.111^***^		−0.178^***^	−0.128^***^
		(0.019)	(0.019)		(0.023)	(0.023)		(0.026)	(0.026)
Primary enrollment (100,000)t-1		−0.001^***^	−0.001^**^		−0.002^***^	−0.001^***^		−0.002^***^	−0.002^***^
		(0.000)	(0.000)		(0.000)	(0.000)		(0.000)	(0.000)
First 5 years of peace			0.063^***^			0.082^***^			0.095^***^
			(0.012)			(0.014)			(0.016)
Health Exp.(% Govt. Spending)t-1			−0.001			−0.001			−0.002
			(0.002)			(0.002)			(0.002)
Constant	3.370^***^	3.376^***^	3.505^***^	4.063^***^	4.062^***^	4.211^***^	4.395^***^	4.387^***^	4.550^***^
	(0.025)	(0.028)	(0.041)	(0.031)	(0.034)	(0.050)	(0.035)	(0.038)	(0.057)
Observations	1343	997	859	1351	997	859	1351	997	859
Groups	73	70	70	73	70	70	73	70	70
*F-Stat*	79.99	56.75	41.89	87.41	57.69	40.67	89.30	56.53	38.71
*Probability > F*	0.000	0.000	0.000	0.000	0.000	0.000	0.000	0.000	0.000

Mortality Rate is in 1000 live births (logged)

Estimation in fixed effects (within) regression. Standard errors in parentheses

*CPA* Comprehensive peace agreement, *GDP* Gross domestic product

^*^ *p* < 0.05, ^**^ *p* < 0.01, ^***^ *p* < 0.001

From the analysis presented in Table 2, the CPA implementation rate is consistently significant and negatively related to the child mortality rates. This supports the argument that countries that have negotiated and implemented CPA provisions have better performances in reducing neonatal [*p* < 0.05], infant [*p* < 0.001], and under-5 mortality rates [*p* < 0.001]. These findings hold after controlling for various factors likely to impact the overall performance of the health sector, including the government expenditure on the health sector.

Among armed conflict and termination-related controls, rebel victory leads to higher neonatal [*p* < 0.001], infant [*p* < 0.001], and under-5 mortality rates [*p* < 0.001], whereas a termination either through government victory or a peace accord has no significant effect. These findings establish that the positive effects of a peace agreement on child survival rates are evident only when the agreement is comprehensive and its provisions are implemented at a high rate.

The cumulative deaths variable, which captures battle related death, is found to be related to lower neonatal, infant, and child mortality rates. This relationship is consistent across all models and significant [*p* < 0.001]. There is a statistically significant relationship between ongoing conflict and higher infant, neonatal, and under-5 mortality rates [*p* < 0.01]. The territorial conflict variable is not significant. Similarly, there is no statistically significant relationship between war duration and child mortality rates.

Among other control variables, primary school enrollment is found to reduce neonatal, infant, and under-5 mortality rates [*p* < 0.001]. Similarly, the GDP per capita variable is negative and significant across all models, which suggests lower child mortality rates with higher levels of GDP per capita [*p* < 0.001]. A constraint on the executive is found to lower neonatal, infant, and under-5 mortality rates [*p* < 0.001]. The first 5 years of peace variable is positive and significant across all models suggesting that the early post-conflict years are challenging and that recovery in a healthcare system takes time. There is no statistically significant relationship between government expenditure on the health sector and child mortality among post-armed conflict countries.

### Note on endogeneity

An endogeneity problem can exist if the dependent and main explanatory variables are influenced by other factors that are not accounted for in the statistical model. In the analysis performed, the potential influence of regime type is controlled by using the executive constraint variable. Economic factors are also controlled by using the GDP per capita variable. The analysis also captures the influence of various measures related to conflict, including the first 5 years after conflict termination. Levels of education may also influence child mortality rates, in addition to peace agreement implementation. Therefore, the analysis controls for education. After controlling for these factors, the relationship between the CPA implementation rate and neonatal, infant, and under-5 mortality rates still holds.

### Limitation: equity issues in post-war public health

Equity in public health access and the quality of care are critical issues for societies emerging from armed conflict [38, 39]. While empirical analyses performed and presented in this article suggest declines in neonatal, infant, and under-5 mortality rates among post-CPA states with high CPA implementation rates, these findings are not indicative of equity in public health access and quality. As the World Health Organization suggested in its 2011 report, aggregate national level data, which are used in this analysis, can mask inequalities among population subgroups (p. 37) [5]. As such, post-armed conflict states that negotiate and implement CPAs can still achieve significant progress toward MDG 4 (reducing child mortality) at the national level while some of the regions within the country significantly lag behind. Research consistently shows that a higher risk of armed conflict correlates with a higher rate of child mortality [40, 41]. Likewise, post-armed conflict states with higher child mortality rates are more susceptible to the recurrence of armed conflict [42, 43]. In this regard, as CPA implementation is related to a decline in child mortality rates, post-CPA states with higher implementation rates will have a lower risk of relapse into violent conflict. Nevertheless, when healthcare provision and quality is inequitable across a country, unaddressed grievances may still lead to a higher risk of conflict recidivism.

**Fig. 1:**
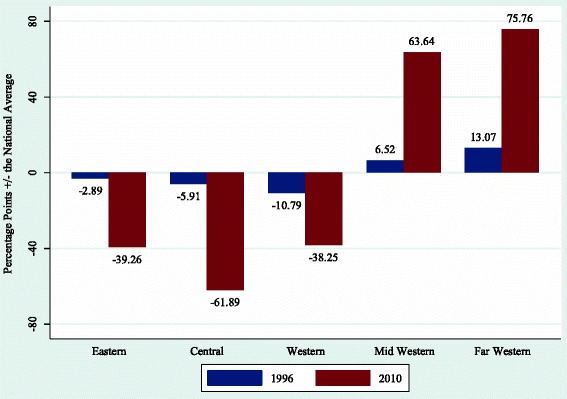
Difference in neonatal mortality rate in 1996 and 2010 across five regions in Nepal

As an illustration, armed conflict in Nepal started from the Mid-Western region of the country which faced relatively low provision and quality of healthcare service compared to the rest of the country. After 11 years of insurgency, a CPA was signed in November 2006, with an implementation rate of 60 % by 2012. Within a few years after the resolution of violent conflict, Nepal achieved its target for the MDG 4 in 2010 [44]. While access to healthcare has improved over the years, the regional disparity that existed prior to the onset of armed conflict has gotten worse. In 1996, 4375 babies were delivered in the presence of health professionals, and by 2010, that number had increased to 326,857. This suggests an improvement in access to healthcare services, even though most of these services were concentrated in urban centers. Figure 1 shows the neonatal mortality rate in five different regions in Nepal, comparing the national average in 1996 and 2010 [44, 45]. As the chart indicates, Eastern, Central and Western regions in Nepal have lower neonatal mortality rates (compared to the national average), both prior to the onset of conflict in 1996 and 5 years after conflict termination in 2010. In comparison, the neonatal mortality rates in the Mid-Western and Far Western regions were higher than the national average in 1996, at 13.07 and 6.52 % respectively. This gap further increased to over 63 and 75 % in the post-CPA period in 2010. One of the reasons for the persistence in regional imbalance is the failure on the part of the Nepali government to send trained doctors to those regions. As a result, many rural communities outside urban centers relied too much on paramedics for basic health services [46]. Even though Nepal met the MDG 4, the regions where the armed conflict started have been neglected by the post-war Nepali government in health provisions. Nepal is illustrative of how an overall decline in child mortality in post-CPA countries does not necessarily represent equal access and quality in healthcare service.

While this article finds that an increase in CPA implementation rate leads to a decline in infant mortality rates, this significant finding should be interpreted cautiously for the following reasons. First, the infant mortality data used in the analysis was macro-level or national level data. For this type of study, it is ideal to analyze micro-level data from those specific communities affected by armed conflict because the national level data may mask community-level health performance. Annualized child mortality data for all conflict-affected communities around the globe however do not exist. Second, data coming from rural villages or remote parts of the country are expected to be faulty, incomplete or non-existent. While faulty data from few communities are less likely to influence the general and aggregate national level data, faulty data from many villages or communities can influence the aggregated national level data. Lastly, due to the lack of annualized data the analysis performed in this study was unable to include indicators measuring the availability of health professionals and their effectiveness in health services delivery. More complete and verified data from household surveys in conflict affected areas will further determine whether there have been improvements in child survival rates in post-CPA states.

## Conclusion

Various studies have shown that countries with high child mortality rates are more susceptible to the onset of armed conflict, and that these conflicts arise in the areas of the country with the highest child mortality rates. Armed conflict also leads to higher conflict-related deaths and injuries in the post-conflict period. Moreover, armed conflict is likely to recur in countries with high child mortality rates. In empirical social science research, child mortality is used as an indicator that best captures variations in socio-economic development. This suggests that armed conflict is both a development and a public health issue. This study empirically demonstrates that the child mortality rate declines more rapidly in post-armed conflict countries that negotiated and implemented CPAs than those that did not.

Post-CPA countries face short-term and long-term needs related to health provisions. In the short-term, the cessation of hostilities after the CPA is signed often leads to a rapid return of IDPs and refugees to their prior communities. Many of these communities, devastated by the conflict, lack physical health infrastructure and trained health professionals to provide critical assistance in safe motherhood, child immunization, child malnutrition, and other preventative care needs. Access to food and safe drinking water are equally critical. These issues can be addressed in the short term when voluntary communities of health professionals can access and work in these war-affected communities. Donor countries and agencies often provide the critical support for this work. If short-term medical needs are met, the child mortality rate may be less likely to increase. After a CPA is signed, armed hostilities cease and former warring groups gradually return to normal activities, a different result than with a decisive government or rebel victory. CPA negotiation and implementation signals a commitment to peace from former warring parties. Donor countries and agencies may consider active CPA implementation in a post-armed conflict country as a sign for readiness and a higher likelihood for success in their intervention plans. As suggested in this article, an improved public healthcare sector will decrease child mortality rates in the long term. The performance of the healthcare sector can significantly improve through better policies and structures negotiated in a CPA. Many provisions in CPA seek to address prior grievances which led to the onset of the conflict, including a variety of political, social and economic realities, often having a direct or indirect impact on healthcare provision in the country. While CPA provisions seek to address these grievances, robust monitoring, evaluation, and advocacy will be needed to ensure and measure the success of addressing grievances through CPA implementation.

In the long term, implementing provisions negotiated in CPAs can help address grievances related to political, social, and economic policies. Addressing these grievances will significantly influence the overall public healthcare sector and its performance alongside other sectors. An improved public healthcare sector will decrease child mortality rates in the long term. The critical issue, however, is whether institutional reforms and new policies will meaningfully address the underlying grievances of the masses. Robust monitoring, evaluation, and advocacy help to understand the extent underlying grievances are addressed when CPA provisions are implemented. Donor countries and agencies, as well as monitoring and advocacy groups, can provide valuable resources and inputs in this regard.

### Ethical approval

Ethics Approval/Statement EA not required. This manuscript does not use data collected from human subjects. Article provides sources for the data used in the analysis.

## Additional file

Additional file 1:
**List of post-armed conflict countries.** (DOCX 14 kb)

## Abbreviations

CPA: Comprehensive peace agreement
PAM: Peace Accords Matrix
UCDP: Uppsala Conflict Data Program
IDPs: Internally displaced persons
MDG: Millennium Development Goals
UNICEF: United Nations International Children’s Fund
GDP: Gross domestic product

## Footnotes

1 In this article, “child mortality rate” refers to neonatal, infant, and under-the-age-of-five mortality rate. Neonatal mortality rate measures the number of those who die before reaching 28 days of age (out of 1000 live births); infant mortality rate measures the number of infants dying before reaching 1 year of age (out of 1000 live births); under-5 mortality rate measures the probability that a newborn baby will die before reaching age five (out of 1000 newborns).

2 The UCDP defines a situation as *armed conflict* when at least 25 battle-related deaths (including military and civilian fatalities) occur in one calendar year. *Armed conflict termination* is coded when fighting stops in a conflict dyad for more than a year either by signing an accord, a decisive military victory (rebel or government), or no/low conflict activities. If a country is fighting multiple conflicts and one conflict is coded terminated, a post-armed conflict year starts.

3 Conflict termination data is updated up to 2012.

4 *Minimum* implementation means that signatories of the accord have made some efforts toward implementation, but the current rate of progress, if continued, will not produce full implementation. *Intermediate* implementation means that the current efforts and rate of implementation are viable to produce full implementation in two-three years. *Full* implementation refers to complete or nearly complete implementation processes.

5 Terminated in peace agreement variable is coded when the conflict is coded terminated either through (1) peace agreement, (2) ceasefire agreement with conflict regulation, or (3) ceasefire agreement.
