# Supplementary material for: Comprehensive peace agreement implementation and reduction in neonatal, infant and under-5 mortality rates in post-armed conflict states, 1989–2012
Source: BMC Int Health Hum Rights. 2015 Oct 8;15:27. doi: 10.1186/s12914-015-0066-7 (PMC4598964; doi:10.1186/s12914-015-0066-7)
Supplement: Additional file 1: — List of post-armed conflict countries. (DOCX 14 kb) [file 12914_2015_66_MOESM1_ESM.docx]

| List of post-armed conflict countries | | |
| --- | --- | --- |
| Afghanistan | Indonesia^*^ | Paraguay |
| Angola^*^ | Iran | Peru |
| Azerbaijan | Iraq | Philippines^*^ |
| Bangladesh^*^ | Israel | Romania |
| Bosnia-Herzegovina^*^ | Ivory Coast^*^ | Russia (Soviet Union) |
| Burundi^*^ | Laos | Rwanda^*^ |
| Cambodia (Kampuchea) ^*^ | Lebanon^*^ | Senegal^*^ |
| Central African Republic | Lesotho | Serbia (Yugoslavia) |
| Chad | Liberia^*^ | Sierra Leone^*^ |
| China | Libya | Somalia |
| Comoros | Macedonia, FYR^*^ | South Africa^*^ |
| Congo^*^ | Mali^*^ | Spain |
| Croatia^*^ | Mauritania | Sri Lanka |
| Djibouti^*^ | Mexico | Sudan^*^ |
| DR Congo (Zaire) | Moldova | Tajikistan^*^ |
| Egypt | Morocco | Timor-Leste^*^ |
| El Salvador^*^ | Mozambique^*^ | Trinidad and Tobago |
| Ethiopia | Myanmar (Burma) | Turkey |
| Eritrea | Nepal^*^ | Uganda |
| Georgia | Nicaragua | United Kingdom^*^ |
| Guatemala^*^ | Niger^*^ | Uzbekistan |
| Guinea | Nigeria | Venezuela |
| Guinea-Bissau^*^ | Pakistan | Yemen |
| Haiti | Panama |  |
| India^*^ | Papua New Guinea^*^ |  |

Note: ^*^Comprehensive peace agreement.
